# Southern Italian teenagers: the older they get, the unfit they become with girls worse than boys: a cohort epidemiological study: The adolescents surveillance system for the obesity prevention project (ASSO)

**DOI:** 10.1097/MD.0000000000008810

**Published:** 2017-12-22

**Authors:** Monèm Jemni, M. Justin Zaman, Daniela La Rocca, Garden Tabacchi

**Affiliations:** a Department of Sport Science, College of Arts and Sciences, Qatar University, Doha, Qatar; b James Paget University Hospital, Gorleston-on-Sea, Norfolk, UK; c Department of Sciences for Health Promotion and Mother Child Care “G. D’Alessandro”, University of Palermo, Palermo, Italy.

**Keywords:** adolescents, age, anthropometric measures, fitness tests, gender, obesity

## Abstract

Italy comprises a high proportion of people who never exercised. Low physical activity levels in adolescents is a risk factor for several disorders. The aim of this cohort epidemiological study was to compare physical fitness profiles between boys and girls with regard to age and gender and to identify health and fitness-related markers that contribute to the make-up of Southern Italian teenagers.

Eight hundred eleven teenagers were assessed for anthropometric measurements and completed the 5 ASSO-fitness tests battery. Data were analyzed with a 2-way analysis of variance (ANOVA) for repeated measures to compare the effect of both age and gender on the fitness components.

The boys’ anthropometric measurements were superior than the girls as expected [weight, height, body mass index (BMI), and waist circumference]; the overall BMI was found in the normality range. The overall teenagers’ fitness markers were found to be quite poor with the boys outperforming the girls in all fitness tests. The weak cardiorespiratory performance of the female teenagers was remarkable. The under 16 years old (-16 yrs) girls outperformed the over 16 years old (+16yrs) girls. There were less significant differences when comparing (-16) and (+16) yrs old mixed-gender groups. There were no correlations between the (−16) and (+16) yrs when both genders were considered. The trend analysis showed the younger teenagers might be “catching up” the older ones in both contexts.

Gender significantly influenced all variables. Although age did not influence cardiorespiratory fitness, the older the teenagers the worse their health and fitness markers become with the older girls worse than their younger peers.

## 1 Introduction

Low physical activity levels in youth are health risk factors, which are predictive of several disorders such as obesity. These disorders could have adverse future consequences on premature mortality and morbidity in adulthood.^[1,2]^ There has been a worldwide increase in obesity among younger people over the last few decades associated with inadequate levels of physical activity.^[3]^ Investigations on people from the European Union's (EU) 28 Member States showed that the majority (39–42%) never exercise and/or never engage into regular or nonregular sport/physical activities, while only (8–9%) exercise regularly.^[4]^ EU reports have shown shocking rates of children's engagement with sport and/or exercise: 68% of the under 15 years old never exercised at the end of their education cycle. This figure is kept significantly high between the ages of 16 and 19 years (45%).^[5]^ Southern European countries seem to comprise the highest rate of people who never exercise. Italy has one of the lowest rates of adolescents meeting current guidelines of regular moderate-to-vigorous physical activity (10.7% compared with 37.6% in Ireland between 2002 and 2010).^[6]^ The lack of interventions aiming to increase the above rates at an early age would result in poorer outcomes. Interventions in this area are widely different; Asides from focusing on behavior-change (dietary intake, physical activity, sedentary behaviors such as watching television, etc.), the actual monitoring of weight-related behaviors is also important.^[7]^

Many countries have recognized the importance of the full assessment of physical fitness at an early stage and have included strategies to do this in their education systems.^[8–10]^ The ASSO project has been funded by the Italian Ministry of Health and supported by the World Health Organization (WHO). It aimed to create a new surveillance system that monitors adolescents’ health and lifestyles. ASSO project was piloted in the city of Palermo's high schools (Southern Italy) and has set out a standardized database of variables, such as anthropometrics, physical fitness, diet, drinking, and smoking habits.

This paper aimed to compare the physical fitness profiles of Southern Italian teenagers and investigating the influence of age and gender. We ultimately aimed to identify health and fitness related markers that contribute to their make-off.

## 2 Methods

This study is a cohort epidemiological study with a cross-sectional assessment. The method section is designed according to STROBE criteria.

### 2.1 Participants

A total number of 811 school pupils (504 boys and 307 girls) participated in this study. All subjects were recruited from year groups 1, 2, 3, and 4 (ages 13–19) from 7 high schools within the city of Palermo in Italy, representing 13.46% of the city’ schools. All schools’ types were represented in this sample (public, private, general education, professional schools, and different socioeconomic areas).

Ethics released was obtained from the ethical committee of the Azienda Ospedaliera Universitaria Policlinico “Paolo Giaccone” in Palermo (approval code n.9/2011). The study was undertaken in accordance with the deontological norms laid down in the Helsinki Declaration (Hong Kong revision, September 1989) and the European Union recommendations for Good Clinical Practice (document 111/3976/88, July 1990). Parents and guardians have been given a full information pack explaining the details of the study and written consent forms have been requested in order to participate in the study. Before any assessment, each participant was assessed for any health issues or contraindications for exercise testing via a health and fitness questionnaire. The entire study took place between January and December 2013.

### 2.2 Assessments and procedure

Weight, height, and waist circumference were assessed using calibrated scales, stadiometers, and nonelastic meters, respectively available in the schools. Participants performed the ASSO Fitness Test Battery (ASSO-FTB) that was set following a systematic review of the past and most recent literature^[9]^ and in consultation with experts in the field. The ASSO-FTB contains 5 health-related physical fitness tests, as per the following order: hand-grip test (HG) to assess upper body maximal strength; standing broad jump test (SBJ) to assess lower body maximal power; sit-up test to exhaustion to assess muscular endurance (SU); 4 x 10m shuttle run test (4 x 10 m SRT) to assess speed and agility; and 20-m shuttle run test (20 m SRT) to assess endurance/aerobic capacity. All tests were performed 3 times and the best score was retained for examination, except for the sit-up test and the 20-m SRT, which were performed only once, as they take participants to exhaustion. All these tests were standardized and internationally adopted for many years. Their validity and reliability are not questionable, as they have been widely used, accepted, and published by the worldwide scientific community. For the purpose of this study, the ASSO field tests have gone through a particular scrutiny. A systematic review was undertaken and published in 2015 to determine reliability and usefulness of these field-based tests for the assessment of physical fitness in this particular adolescence age group.^[9]^

After collecting the measurements, all data were entered in the ASSO-NutFit software, to obtain a standardized excel database.

### 2.3 Data analysis

All data have been made anonymous by replacing the names with IDs that only investigators have access to and stored within a secured computer in the laboratory. Students who did not complete the entire anthropometric and fitness tests have been deleted from the database. Normality of distribution was assessed by Shapiro–Wilk tests. Data were presented in mean and standard deviation (SD) when normally distributed and/or medians and range when they were not normally distributed. Homogeneity of the variance was assessed by Levene test and effect size (Es) was established using partial Eta Squared. Comparison between groups was conducted via an independent *t* test for normally distributed data or via a Mann–Whitney test for not normally distributed data. Similarly, and depending on the normality of the distributions, either a parametric (Pearson correlation) or nonparametric (Spearman Rho) test was applied allowing a regression analysis between the fitness tests results. The magnitude of effects was qualitatively assessed according to Hopkins^[11]^ as follows: trivial *r* < 0.1, small 0.1 < *r* < 0.3, moderate 0.3 < *r* < 0.5, large 0.5 < *r* < 0.7, very large 0.7 < *r* < 0.9, nearly perfect *r* > 0.9 and perfect *r* = 1. A correlation-effect size was assessed with coefficient of determination (*R*^2^).

Further comparative analysis was conducted using a 2-way analysis of variance (ANOVA; age and gender) (or a Freedman ANOVA when nonparametric observations) for repeated measures to assess their effect on the 5 considered fitness variables. The level of significance was set at a *P* value ≤ .05 for all the above analysis. All analysis was conducted using Windows Microsoft Excel and IBM SPSS version 20, Armonk, NY, IBM Corp. USA.

## 3 Results

### 3.1 Normality of distribution and homogeneity

Shapiro–Wilk tests for normality of distribution showed all combined boys and girls’ data to be not normally distributed (*P* ≤ .05) for each variable. Data were not normally distributed for boys when split in age group, except for the −16's height and SBJ, as well as, for the +16's HG tests (*P* > .05). The female split age group data were not normally distributed, except for −16's height and SBJ, and +16's SBJ (*P* > .05). The Shapiro–Wilk test showed that the overall boys groups data were not normally distributed except for the HG and the SBJ tests. When considering the overall girls, only height and SBJ test were normally distributed.

Boys were significantly taller, heavier, had a higher BMI, and larger waist circumferences than girls at (*P* < .05). (Table 1). These results were supported by very large effect sizes (Es range; 0.960–0.998). Only the age of the boys and the girls did not significantly differ. When divided into age groups (−16 and +16 years), the older boys were significantly taller and heavier than their youngsters (*P* < .05). Their BMI and waist circumferences were not significantly different (Table 2, section 1). These outcomes were also supported by very large effect sizes (range: 0.957–0.998).

**Table 1:**
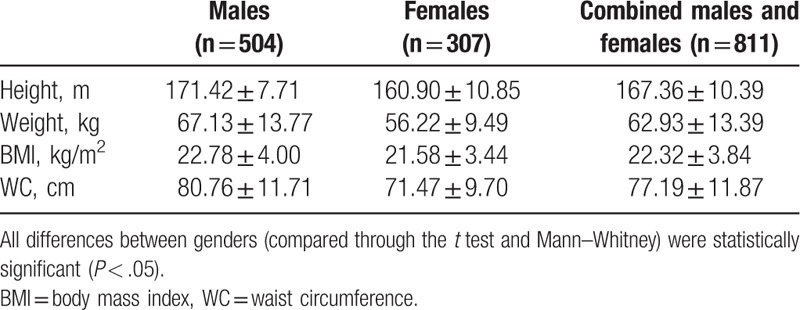
Anthropometric characteristics (means + SD) of the ASSO Project participants, by gender.

**Table 2:**
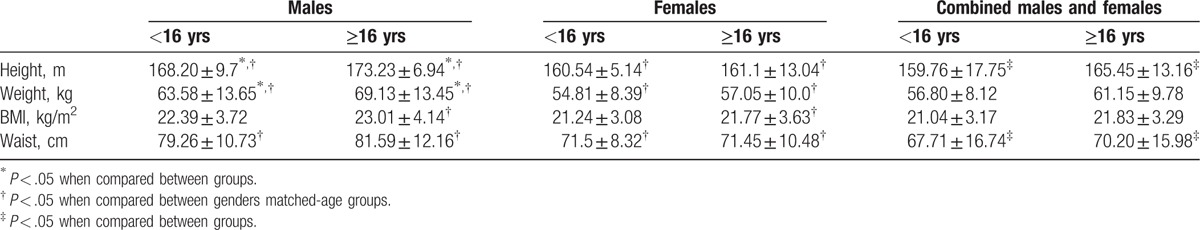
Anthropometric characteristics of the ASSO project participants, by gender and age (means ± SD).

### 3.2 Anthropometric measurements

Comparison between the girls’ −16 and +16's anthropometric data did not show any significant difference (*P* > .05) (effect size range 0.973–0.995) (Table 2, section 2).

The +16 yrs mixed gender group was significantly taller and had larger waists than their −16 yrs old peers (*P* < .05) (Table 2, section3).

When the 2 factors “gender and age” were considered, the height, weight, and waist of the −16 yrs boys were found to be significantly greater than the same age female group (Table 2, section 1 and 2) (*P* ≤ .05). The older male adolescents were found to be significantly taller, heavier, and had higher BMIs and waist circumferences than their similar age female group (Table 2, section 1 and 2) (*P* ≤ .05).

### 3.3 Fitness tests

Fitness tests results of the total sample and/or stratified by age and gender are summarized in Table 3.

**Table 3:**
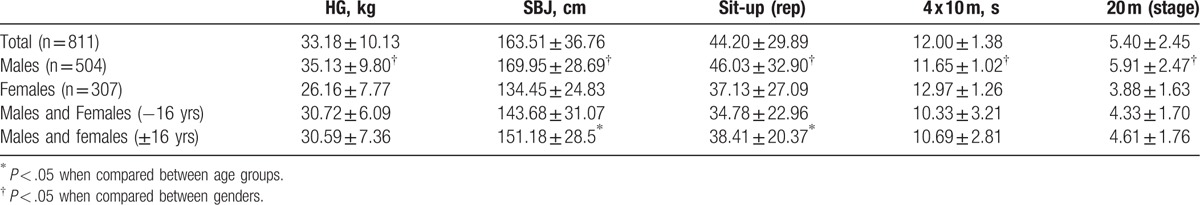
Fitness tests results by gender and by age.

**Figure 1:**
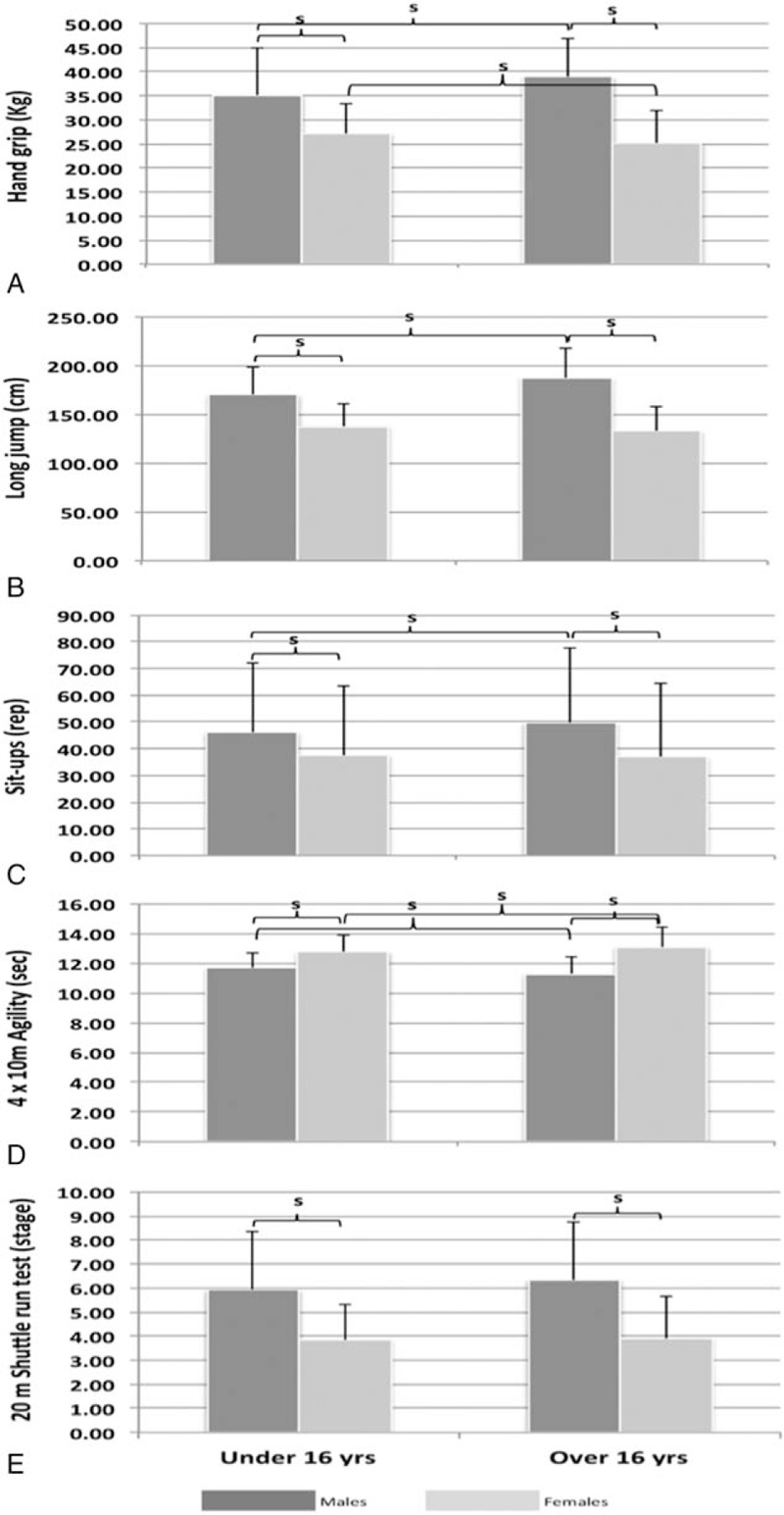
Boys and girls ASSO-FTB results per age and gender groups.

The 504 male teenagers significantly outperformed their peer girls at all fitness tests (*P* < .05). The 2-ways ANOVA (age and gender) showed that both under and over 16 yrs old boys significantly outperformed their peer female group (*P* < .05, Fig. 1A–E).

#### 3.3.1 Handgrip test

Results showed that the boys were stronger than the girls in the handgrip test [*P* < .05 and supported by a very large Es (0.935)], Table 3. The −16 years olds mixed group of boys and girls showed similar performances at this test when compared with the older mixed-gender group (30.72 ± 6.09 vs 30.59 ± 7.36 kg, *P* > .05, Table 3). When divided into age groups, the +16 years old boys had significantly higher handgrip values when compared to the −16 yrs (38.9 ± 9.8 vs 35.15 ± 8.06 kg, respectively, Fig. 1A) (*P* ≤ .05; Es = 0.944). However, younger girls performed significantly better in this test than their older peers (27.13 ± 6.29 vs 25.19 ± 6.50 kg respectively, *P* ≤ .05; Fig. 1A).

#### 3.3.2 Broad jump test

The boys were again significantly better in this test compared with the girls (*P* < .05); the difference was supported by a very Es (0.827), Table 3.

The +16 years old mixed gender group of boys and girls jumped significantly longer distances than the younger mixed-gender group (*P* < .05, Table 3). When divided into age groups, the +16 years old boys jumped significantly longer than the −16 yrs (187.55 ± 30.99 vs 169.95 ± 28.69 cm, respectively; *P* < .05; Fig. 1B) (ES = 0.971). In contrast, the older girls did not score as well as their younger peers (132.46 ± 25.69 vs 137.89 ± 22.98 cm, respectively, Fig. 1B); we should mention that this difference did not reach a statistical significance (132.46 ± 25.69 vs 137.89 ± 22.98 cm, respectively, Fig. 1B).

#### 3.3.3 Sit-up test

Boys performed significantly more sit-ups than girls [(*P* < .05); ES = 0.965, Table 3].

The +16 years old mixed gender group of boys and girls performed more sit-ups than the younger mixed-gender group (*P* ≤ .05, Table 3).

When stratified into age groups, the +16 years old boys performed significantly more sit-ups than the −16 years old (49.81 ± 29.38 vs 46.03 ± 32.90 respectively; ES = 0.693; *P* ≤ .05, Fig. 1C). However, the older girls performed slightly less sit-ups compared with their younger peers; this difference was not statistically significant either (36.91 ± 27.73 vs 37.55 ± 25.96, respectively; ES = 0.631; *P* > .05; Fig. 1C). Worth to note the highs standard deviations in both cases.

#### 3.3.4 4 x 10 m shuttle run test

Boys performed significantly better than girls in this speed and agility test (Table 3).

The +16 years old mixed gender group of boys and girls performed similar performance to the younger mixed-gender group, *P* > .05 (Table 3).

When divided into age groups, the +16 years old boys performed significantly better than the −16 years group (11.28 ± 1.11 vs 11.65 ± 1.02 seconds, respectively; Fig. 1D). However, girls −16 years of age were significantly quicker than their peers aged +16 years (12.76 ± 1.13 vs 13.09 ± 1.32 seconds, respectively; Fig. 1D).

#### 3.3.5 20 m shuttle run test

The analysis of the 20 m shuttle run test performance showed that although the 504 boys performed significantly better than the 307 female teenagers [5.91 ± 2.47 vs 3.88 ± 1.63 stage, (*P* < .05), Table 3], the entire group's performance was quite weak. Surprisingly, the +16 yrs old mixed group of boys and girls performed similarly to the younger mixed-gender group (*P* > .05) (Table 3).

When divided into age groups, the +16 years old boys did not significantly outperform the −16 years (6.33 ± 2.41 vs 5.91 ± 2.47 stages, respectively; *P* > .05; Fig. 1E) and similarly when comparing the −16 years old girls to their peers +16 years (3.90 ± 1.74 vs 3.85 ± 1.45 stages, respectively; *P* > .05; Fig. 1E).

### 3.4 Correlation analysis

#### 3.4.1 Anthropometric measurements

A strong and significant correlation was noticed between the subjects’ weights and their waist circumferences when examining the entire group and also when considering each age category for each gender (Table 4). Furthermore, a strong and significant correlation was found between the subjects’ BMIs and weights in all groups’ categories. Similar strong correlations were also found between the BMIs and the waists in all categories.

**Table 4:**
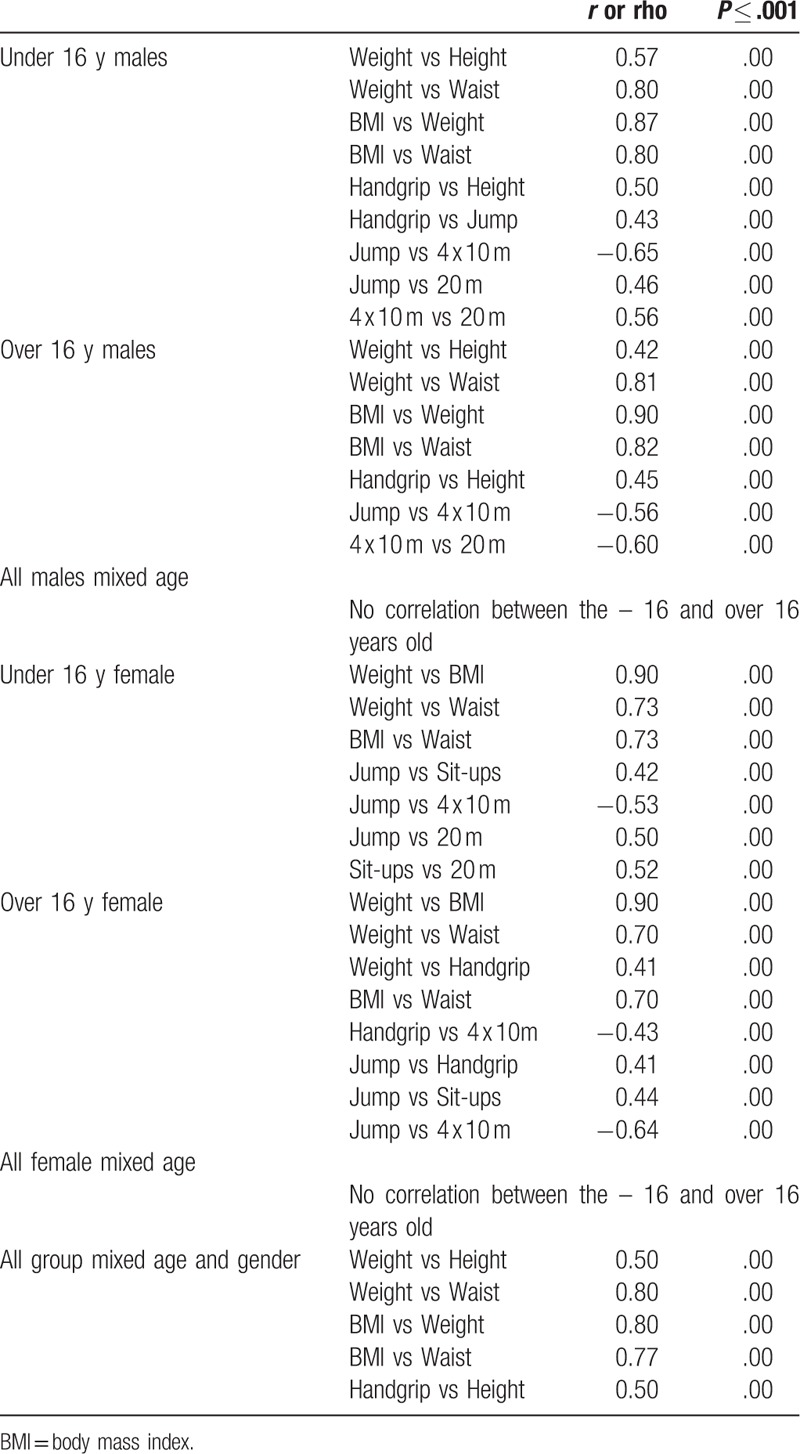
Significant correlations between the variable.

There were no significant correlations between anthropometric variables when considering the boys’ age categories (−16 vs +16 yrs). Similar results were found when correlating the under 16 to the over 16 years’ girls.

#### 3.4.2 Fitness tests

A multi-regression analysis was carried out between the entire performances in each gender and/or in each age and gender together (Table 4). There was no correlation between the −16 and the +16 yrs old in either genders.

Some significant large correlations were noticed within each combined age and gender category, with *r* and/or Rho was between 0.5 and 0.69 (Table 4). For instance, the long jump test was negatively correlated to the 4 × 10 m shuttle run in all combined age and gender categories. However, not all relations had similar outcomes between the boys and girls. The 4 x 10 m agility test was negatively correlated to the 20 m shuttle run test within each age category male groups. In addition, the handgrip test was significantly correlated to the heights of the boys, but both relations were not statistically significant within the female groups (Table 4).

## 4 Discussion

This paper investigates the influence of age and gender on the anthropometric characteristics and physical fitness performances of a significant sample of adolescents from Southern Italy. Our focus was to identify any trends and/or differences between the younger and the older teenagers in the hope to tackle any related issues from sources.

Although the entire sample's BMI was found to be in the normal range, the findings indicated that boys were significantly taller, heavier, and had higher BMIs and larger waist circumferences than similarly aged girls. The fact that the BMIs and the waist circumferences of the 2-age male groups did not differ would lead us to ask the following question: Are the young teenagers boys outsizing the older ones, or is it the opposite? The lack of any correlations between the −16 and the +16 years in both genders would actually suggest this fact. Further analysis showed that the −16 BMI and waist circumferences’ incline trends were slightly higher with a nonsignificant regression coefficient, compared with the +16 yrs. Consequently, these analyses confirm that the younger boys’ BMI and waists circumferences are in very slow increase patterns, whereas the older boy's variables are almost stable. This main result strengthened the EU reports that showed children's engagement with sport and/or exercise is shocking: 68% of the under 15 years old never exercised at the end of their education cycle. This figure is indeed kept significantly high between the ages of 16 and 19 years (45%).^[4]^

More importantly, the girls’ body anthropometric data are to consider very carefully because there was no significant difference between the younger and the older teenagers. This result again confirms the same trend shown with the boys and suggesting that the younger teenagers girls might be “catching up” with the older ones.

Some of the main findings of the fitness tests were obvious, such as, the significant differences between male's fitness scores over those of the girls. Physiologically, boys have indeed greater overall muscle mass, larger muscle, and larger muscle fibers than girls^[5]^; this may explain these differences. However, the overall fitness performances were quite similar to the ones showed in a European sample of more than 3000 people collected within the HELENA study,^[12]^ respectively: HG (33.18 ± 10.13 vs 31.2 ± 9.1); SLJ (163.51 ± 36.76 vs 164.7 ± 35.5); 4 × 10 SR (12.00 ± 1.38 vs 12.2 ± 1.4); 20 m SRT (5.40 ± 2.45 vs 5.0 ± 2.7).

Our study shows larger variations in the fitness scores when comparing the elderly to the younger groups; For instance, the +16 yrs old girls achieved a score of 25.19 kg in the handgrip test, whereas the +16 boys achieved 38.92 kg, which is a larger variance than within the younger groups (27.13 and 35.15 kg, respectively). Abernethy et al^[13]^ tried to explain this variation by the fact that up to the age of 13 years old, boys and girls have relatively similar muscle mass and bone density. It is also known that the onset of adolescents, hormonal influences within males, causes greater increase in bone and muscle mass than girls.^[5]^

Some other findings were quite concerning, such as, the lack of significant differences and/or correlations between the age groups. It was expected that the +16 years old outperform their −16 years’ peers in each gender; however, some of the findings were in somehow remarkable and would even require particular attention from the local health authorities.^[14]^ Being taller and heavier, the +16 years old boys performed statistically greater scores in the handgrip strength, long jump, 4 × 10 m shuttle run test, and number of sit-ups. However, this was not the case when comparing the cardiorespiratory endurance assessed by the 20 m shuttle run test, where no statistically difference was noticed. Surprisingly, the younger girls significantly outperformed their older peers in the handgrip and the agility test (4 × 10 m) (27.13 vs 25.19 kg and 12.77 vs 13.09 seconds, respectively). We must add that the same younger girls performed few more sit-ups than the older girls without reaching a significant difference. Similar to the boys, the cardiorespiratory fitness of the older and the younger girls did not statistically differ, highlighting unchanged aerobic ability even with age difference. Unquestionably, these results confirm the following statement: “as the teenager girls get older, the unfit they become.” One of the explanations that has been suggested by the European reports is the fact that the older the teenagers, the more they drop out from the physical education sessions and from other physical activities.^[4]^

An average of 5.91 ± 2.47 stages at the 20 m shuttle run test was showed in all age males, whereas the girls showed an average stage of 3.88 ± 1.63 only. The figures are considered even poorer when combining both genders and considering the age categories; in that, the −16 yrs old mixed-gender group performed 4.33 ± 1.70 stages compared with the older mixed-gender group who performed 4.61 ± 1.76 stages. It is generally admitted that girls are less active than boys at a same age.^[15]^ The transition from primary school to high school is associated with several pressures not only at home but also at school and outside. It is also generally thought that teen girls are more concerned about their body image, the onset of menstruation, and the associated state of mind, the self-confidence, and the insecurity about the changes occurring in those first years of teenager-hood. For these reasons, physical activity takes a back seat to other priorities. Teen boys, however, have less concerns about the changes and would generally increase their activity levels. It is also admitted that during postpuberty, the boys would gain a significant boost in strength and power thanks to the sexual hormone testosterone. This fact is indeed confirmed in our study seeing the older boys have outperformed their young peers in the handgrip strength, the power test, the abdominal stamina test, and the agility test. However, the cardiorespiratory fitness was not different, which leads to questioning the age groups activity levels and nature. The ASSO toolkit has surveyed this item among the teenagers as well as their parents’ exercise per week. Surely, a correlative investigation would provide some answers in this context and could be the object of further publications.

As a summary, our study highlighted issues related to the anthropometric measurements of teenagers, with the young ones tending to outsize the older ones. This finding was also associated with an overall decrease in the fitness level, as the teenagers become older with a more significant gap between the younger girls and their peer older ones. We hope that these findings would not only trigger further investigations in other communities of different socioeconomic features but would also encourage the local government to spark a suitable action plan. Schools’ physical education hours for instance should be increased rather than decreased, as it is currently the case in several EU countries. Schools head-teachers should discourage swapping physical education hours with more lectures at examination periods. It is also important to mention that this cohort study does not represent the entire Italy, as there are some differences in wealth and resources between the South and the North for instance, which also means different lifestyle, diet, and activity level.

## 5 Limitations

Our data collection methods could easily be replicated by teachers with the exception of the handgrip test, which is generally not available within the typical school environment and moreover not usually often affordable by the schools. In this study, the handgrip was provided by the ASSO team and this created some difficulties in the autonomous management of the system. Moreover, the study sample was from a single city, thus reducing the generalizability of the results to a larger population or populations from rural or mountain areas and small towns. Another limitation is that the sample was composed of a higher number of male adolescents than girls; this was due to the sample stratification per type of school, which did not take into account the gender composition of each school in the initial part of the study. Finally, not all teachers were available to participate in the data collection or wanted to contribute to the project, which limited the success of the project in some schools.

On the opposite side, we shall mention that this study has some strength, such as the good sample size, the good adherence of the participants and the limited number of drop out, the engagement of different types of schools, and the engagement of school teachers in data collection and data entry.

## 6 Conclusion

The aim of this study was to compare physical fitness profiles between boys and girls with regard to age and gender and to identify health and fitness related markers that contribute to the make-up of Southern Italian teenagers. Our study highlights that gender influences body and fitness measurements in a sample of adolescents, with boys showing higher anthropometric measurements and significantly higher performances than similarly aged girls in all the fitness tests. These differences were not significant when comparison involved under and over 16 years old mixed-gender groups for some of the fitness variables, including cardiorespiratory component. The trend analysis showed that the younger teenagers might be “catching up” with the older ones in both contexts. Hence, the younger ones could be similar to the elders if their situation does not change. Although age did not influence cardiorespiratory fitness, the older the teenagers, the worse their health and fitness markers become, with the older girls worse than their younger peers. These variables could be affected by their sedentary lifestyle and the lack of exercise mentioned in European reports since 2014. The findings could help the local government to initiate some plans encouraging teenagers (girls in particular) to become more active and to reenforce obesity prevention.

## Acknowledgment

The authors would like to acknowledge Mr Mohammad Shoaib Prince for his help in formatting and in the submission process of the manuscript.
